# Adaptive Vibrational Coordinates *via* Symmetry-Aware Normalizing Flows

**DOI:** 10.1021/acs.jctc.6c00224

**Published:** 2026-06-04

**Authors:** Emil Vogt, Álvaro Fernández Corral, Yahya Saleh

**Affiliations:** 1 155358 Center for Free-Electron Laser Science Cfel, Deutsches Elektronen-Synchrotron DESY, Notkestr. 85, Hamburg 22607, Germany; 2 Independent Researcher, Hamburg, Germany; 3 Independent Researcher, Madrid, Spain

## Abstract

Normalizing flows have recently been proposed to learn optimal vibrational coordinates tailored for a given molecule. It was demonstrated that such learned coordinates achieve substantial improvements in the accuracy of vibrational spectral calculations and capture essential vibrational behavior of molecules. We extend this framework to incorporate molecular symmetry by introducing a novel *G*-equivariant invertible residual network architecture. Exact equivariance with respect to a chosen discrete molecular symmetry group is enforced by construction, ensuring that the learned coordinate transformation respects the imposed symmetry for all network parameters. When combined with a symmetry-adapted basis, this equivariance preserves the symmetry-induced block-diagonal structure of the Hamiltonian throughout the optimization. We show that optimizing the coordinates either for a set of states spanning multiple irreducible representations or for a subset transforming according to a single irreducible representation yields comparable results. This result suggests that optimal coordinates are shared across irreducible representations and provides insight into how optimized coordinates accelerate basis convergence. The utility of symmetry-aware normalizing flows is demonstrated for H_2_CO and NH_3_.

## Graphical abstract

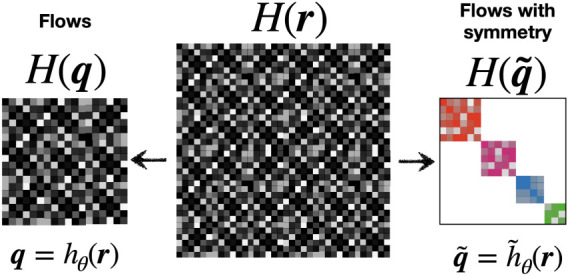

## 1 Introduction

Accurate spectral solutions of the rotational-vibrational Schrödinger equation require an appropriate choice of vibrational coordinates. For a chosen basis, the coordinate set dictates the efficiency of the spectral calculation. An effective set of coordinates must balance several competing factors. It should enable rapid basis convergence, yield easily interpretable results, incorporate molecular symmetry, allow for effective evaluation of the Hamiltonian operator, respect the physical boundary conditions, etc. Traditional geometric coordinate choices, such as rectilinear normal, curvilinear valence-bond, or Jacobi coordinates, offer only limited flexibility in simultaneously satisfying these criteria.^1–8^

Selecting optimal vibrational coordinates for a given molecule typically requires significant intuition and detailed knowledge of the underlying potential energy surface (PES). Since nuclear motions vary widely across molecular systems, no universally optimal coordinate system exists. A promising strategy for addressing this challenge is to introduce a learnable coordinate transformation, i.e., a parametrized mapping that can be optimized according to a predefined objective. Coordinate transformations based on combinations of normal or curvilinear coordinates have demonstrated notable improvements in the accuracy of spectral calculations.^8–18^ However, the broader application of coordinate optimization in rotational-vibrational calculations remains limited. Most prior investigations have focused on linear transformations with inherently limited expressivity.

The choice of coordinates is especially critical when describing highly delocalized vibrational states.^19–25^ If the coordinates induce strong coupling between vibrational modes, the traditional approximations used in spectral methods become inaccurate. Delocalized vibrational states arise not only in floppy molecules, such as weakly bound complexes without a well-defined equilibrium geometry, but also more generally in highly-excited states. Although delocalized vibrational states extend over large regions of configuration space, they can often be described effectively using localized coordinates. The historical success of localized curvilinear coordinates for describing highly excited XH-stretches (where X = O, N, C, etc.) underscores the importance of choosing suitable coordinates to decouple delocalized motions.^26–31^ However, such localized coordinates are beneficial only for specific types of vibrations, and do not generalize easily to other types of vibration.

We have recently introduced normalizing-flow^32–34^ vibrational coordinates,^35,36^ a class of adaptive coordinates that can be learned to optimize basis performance for a given molecule. These coordinates are obtained by applying a flexible, nonlinear, invertible transformation—represented by an invertible neural network, i.e., a normalizing flow—to a set of reference coordinates, such as normal-mode or valence-bond coordinates. In our studies,^35,36^ we used invertible residual networks (iResNets)^32^ to model the coordinate transformation. However, other normalizing-flow architectures, such as real-valued non-volume preserving architectures can also be used.^37,38^

Normalizing-flow coordinates represent a fundamentally different approach from the conventional coordinates employed in calculations of vibrational states. Instead of relying on a fixed geometric construction, they can be tailored to the problem at hand through optimization. With appropriate parameter and model choices, normalizing-flow coordinates can reproduce traditional coordinate types, encompassing and extending the possible coordinate choices to describe molecular systems. Normalizing-flow coordinates thereby offer a systematic route for improving coordinate representations.^35–38^ However, our initial formulation of normalizing-flow vibrational coordinates did not incorporate molecular permutation-inversion (PI) symmetry. Symmetry-restricted normalizing-flow coordinates were recently suggested as the next milestone to expand the applicability and accuracy of these coordinates.^39^

Symmetry plays a central role in the computation of rotational-vibrational spectra of molecules.^40–43^ The PI symmetry group of a molecule can be used to enforce a block-diagonal structure of the Hamiltonian matrix and dictates the symmetry-allowed electric-dipole transitions.^44^ It also determines the nuclear spin statistical weights associated with each irreducible representation, thereby identifying states that are forbidden by the Pauli exclusion principle and governing the relative thermal populations of the symmetry-allowed states. Coordinates and basis functions used in spectral calculations must therefore transform consistently under the PI group to make use of these properties. However, symmetry-restricted coordinates and basis functions are not strictly required. Any computational setup that leads to converged results will ultimately lead to eigenstates that respect the molecular symmetries. Thus, imposing symmetry primarily serves to reduce computational costs, enabling calculations that may otherwise be infeasible.

In this work, we develop symmetry-aware normalizing flows that are exactly equivariant under a chosen PI group *G*; that is, for every *g* ∈ *G*, the group action on the transformed coordinates coincides with its action on the input coordinates. To enforce exact equivariance, we introduce a novel *G*-equivariant iResNet architecture in which the residual functions of each block are symmetrized *via* Reynolds averaging. As a result, the learned coordinate transformations inherit the symmetry of the original coordinates for all network parameters.

We demonstrate the approach on formaldehyde (H_2_CO) and ammonia (NH_3_), showing that optimizing the coordinates by minimizing the summed energies of states spanning one or multiple irreducible representations yields comparable performance. This observation clarifies how optimized coordinates accelerate basis convergence. To explain this behavior, we rely on a recently developed analysis of normalizing-flow coordinates applied to Hermite basis functions, which suggests that optimal transformations modify the asymptotic behavior of Hermite functions to better match that of the target states.^45^

Imposing symmetry introduces a physically motivated inductive bias that restricts the space of admissible coordinate transformations, improving both stability and optimization efficiency. Moreover, symmetry-aware normalizing flows preserve the symmetry-induced block-diagonal structure of the Hamiltonian independently of the coordinate parameters. An analogous construction in electronic-structure theory would similarly enforce the required antisymmetry of electronic wavefunctions under particle exchange.

## 2 Theory

### 2.1 Spectral Methods

In spectral calculations, the vibrational Schrödinger equation,

$$H\Psi_{m}=\left(T+V\right)\;\Psi_{m}=E_{m}\Psi_{m}$$

is projected onto a finite set of basis functions $\{\phi_{n}{\}}_{n=0}^{N-1}$ . Here, *H* is the Hamiltonian, *T* is the kinetic energy operator, *V* is the potential, Ψ_*m*_ is the *m*’th eigenfunction and *E_m_* is the associated energy eigenvalue. The eigenfunctions are approximated by the ansatz

$$\Psi_{m}(r)\approx {\mathrm{\Psi ̃}}_{m}(r)=\sum_{n<N}c_{nm}\phi_{n}(r)$$

where **r** denotes the vibrational coordinates and *c*_*nm*_ is the linear expansion coefficient associated with the *n*’th basis function and the *m*’th eigenfunction. In variational calculations, the linear expansion coefficients are obtained by solving the matrix eigenvalue problem,

HC=SCE

where **H** is the Hamiltonian matrix expressed in the basis, **C** is the coefficient matrix, **S** is the overlap matrix (identity for an orthonormal basis), and **E** is the diagonal eigenvalue matrix containing the approximate vibrational energies {*Ẽ*_*m*_}_*m*_.

The convergence of the computed vibrational energies strongly depends on the choice of basis functions and coordinates. To better adapt the basis to the vibrational problem at hand, we proposed in Saleh et al.^35^ to learn basis functions of the form

1 $$\gamma_{n}(q;\theta )=\phi_{n}(q)\sqrt{D}$$

where ϕ_*n*_ are *a priori* chosen basis functions and **q** = *f*_θ_(**r**) is a change of coordinates, with *f*_θ_ modeled by a normalizing flow parametrized by θ. Here,

$$D=|\det(\nabla_{r}\;f_{\theta }(r))|$$

denotes the absolute value of the determinant of the Jacobian, which can also be evaluated in the **q** coordinates.

A normalizing flow is a concept from the machine-learning literature referring to a sequence of differentiable and invertible transformations.^32–34^ The invertibility of *f*_θ_ ensures that the inverse mapping $r=f_{\theta }^{-1}(q)$ exists. The prefactor $\sqrt{D}$ in eq 1 guarantees that the augmented basis functions remain orthonormal whenever the original basis {ϕ_*n*_} is orthonormal. In addition, the augmented basis remains complete as long as *f*_θ_ is a diffeomorphism,^46,47^ a property enforced by the normalizing-flow architecture used in this work (see Section 2.3 for further details).

Hamiltonian matrix elements in the adaptive augmented basis can be written as matrix elements of a modified Hamiltonian expressed in the underlying *a priori* basis. This follows directly from the change of variables **q** = *f*_θ_(**r**). As an example, the potential energy matrix elements take the form

2 $$\begin{array}{ll} V_{nn^{\prime }} & =\int \phi_{n}^{*}(f_{\theta }(r))\sqrt{D}\;V(r)\phi_{n^{\prime }}(f_{\theta }(r))\sqrt{D}\;dr\\ & =\int \phi_{n}^{*}(q)V(f_{\theta }^{-1}(q))\phi_{n^{\prime }}(q)\;dq \end{array}$$

An analogous, though more involved, expression can be derived for the Podolsky form of the kinetic energy operator and its rearranged representation.^35,36^ These relations show that learning an augmented basis of the form given in eq 1 is mathematically equivalent to learning a modified Hamiltonian expressed in the original basis.

To optimize the normalizing-flow parameters θ, we define the loss function

3 $$L_{\theta }^{M}=\sum_{m<M}{Ẽ}_{m}\to \min_{\theta }$$

where *M* ≤ *N* is the selected number of target states. In principle, numerical integration errors for computed Hamiltonian matrix elements may lead to approximate energies below the variational limit. If *M* = *N*, the loss function becomes $L_{\theta }^{N}=\mathrm{Tr}(H)$ , which is computationally tractable as it does not require the evaluation of off-diagonal elements. However, when only few states are of interest, eq 3 is preferred as it focuses the optimization on this specific subset of states.

The normalizing-flow algorithm allows the underlying basis to be defined on any domain on the transformed coordinate **q**, which is then mapped onto the input coordinate domain **r**. However, as exemplified in Vogt et al.,^36^ in general, different types of basis functions cannot be mapped one-to-one by a change of coordinates. For example, since coordinate transformations cannot change the relative ordering of nodes, the harmonic-oscillator eigenfunctions cannot be mapped into anharmonic functions like the Morse-oscillator eigenfunctions. The odd harmonic oscillator eigenfunctions share a node at *x* = 0, whereas the Morse functions do not. Consequently, the choice of the underlying *a priori* basis influences the achievable accuracy for a fixed number of basis functions. We deliberately employ a simple underlying basis to remove the reliance on expert molecule-specific intuition in selecting a suitable basis. Instead, the complexity is shifted from the basis to the learned coordinates themselves.

### 2.2 Symmetrization

Molecular symmetry groups reflect the true (observable) symmetries of molecular rotational-vibrational states. In spectral calculations, symmetry can be utilized to reduce computational costs and to improve the classification of computed eigenfunctions. The cost reduction depends on the employed methodology. For variational calculations, symmetry enables a block-diagonal representation of the Hamiltonian matrix and makes it possible to reduce the size of the integration grid.

The rotational-vibrational Hamiltonian is invariant under the operations of the Complete Nuclear Permutation-Inversion (CNPI) group of the molecule. In practice, however, one often works with a PI group, which contains a closed subset of the CNPI operations. This type of reduction may be necessary if the employed PES does not strictly transform according to the CNPI symmetry, or if the employed coordinates and/or basis functions do not accommodate all the CNPI operations. For any chosen PI group *G* ⊆CNPI, the Hamiltonian remains invariant under its operations,

$$P_{g}HP_{g}^{-1}=H$$

where *P_g_* is the symmetry operator representing the action of *g* ∈ *G* on rotational-vibrational wavefunctions. Equivalently,

$$[H,P_{g}]=0,,\mathrm{for any}\;g\in G$$

Since the Hamiltonian commutes with every symmetry operator, its eigenfunctions transform according to the irreducible representations of *G*,

$$P_{g}\Psi_{m}^{\left(\Gamma \right)}=\Gamma (g)\Psi_{m}^{\left(\Gamma \right)}$$

where Γ­(*g*) is the representation matrix associated with the irreducible representation Γ. Thus, while the Hamiltonian itself is *invariant*, its eigenfunctions transform *equivariantly* under *g*. The basis functions are symmetry-adapted if each basis function also transforms according to an irreducible representation,

$$P_{g}\phi_{n}^{\left(\Gamma \right)}=\Gamma (g)\phi_{n}^{\left(\Gamma \right)}$$

It follows that the Hamiltonian matrix becomes block-diagonal,

$$H=\underset{\Gamma }{⊕}H^{\left(\Gamma \right)}$$

with one block for each irreducible representation.

A symmetry-adapted basis can be constructed from an arbitrary (non-symmetry adapted) basis, {ϕ_*n*_}, by using the projection operator,

$$\phi_{n}^{\left(\Gamma \right)}=P^{\Gamma }\phi_{n}$$

where

4 $$P^{\Gamma }=\frac{l_{\Gamma }}{\left|G\right|}\sum_{g\in G}\chi_{\Gamma }(g)D_{g}$$

Here, *l*_Γ_ is the dimension of the irreducible representation, *χ*_Γ_(*g*) is its character, and *D_g_* is the representation matrix associated with *g*. The resulting symmetry-adapted basis functions, $\phi_{n}^{\Gamma }$ , span the part of the original basis space that transform as Γ. In general, orthonormalization is required to construct an orthonormal symmetry-adapted basis.

To exploit symmetry to reduce the size of the integration grid, each point must map to another point in the grid under the action of the symmetry operators of the PI group. Hence, the symmetry properties of a grid is not of the individual points (unless the particular point is invariant under all relevant symmetry operations), but rather of the collection of points. Let **R** denote the equivariant integration grid, with grid points **r**_*i*_ as columns. Let $T_{g}:r\to r^{\prime }$ denote the operator in the coordinate space associated with *g*. The action of this symmetry operator on **R** is a permutation of grid points

$$T_{g}R=R\Pi_{g}$$

where **Π**_*g*_ is a permutation matrix, acting on the grid indices. Consequently, each grid point belongs to an *orbit* containing all symmetry related points,

$$O(r_{i})=\{T_{g}r_{i}\;|\;g\in G\}$$

These orbits partition the integration grid into disjoint subsets. Within each subset, the action of a symmetry operation is simply to permute its elements. Only one point is required per orbit, as the contribution to the Hamiltonian for points on an orbit are equivalent up to a symmetry factor. The symmetry factor depends on how the integrand transforms under the symmetry operations. For example, matrix elements between basis functions that transform according to the same irreducible representation have unit symmetry factors for each point on an orbit. The maximum size of an orbit is |*G*|, and the size of the grid can therefore be reduced by a factor of 1/|*G*| without losing integration accuracy. Identically, a non-symmetrized integration grid can be expanded by a factor of up to |*G*| by generating all points on an orbit. Following the same logic, the expanded integration grid produces the same accuracy as the non-equivariant one. This illustrates that symmetry is useful even in non-symmetry adapted integration schemes, such as Monte Carlo integration.

For symmetry to be retained during the training of the normalizing-flow coordinates, the transformed coordinates **q** = *f*_θ_(**r**) must transform under the PI group in exactly the same way as the initial (input) coordinates, for all values of the parameters θ. A normalizing flow that satisfies this condition is referred to as a *symmetry-aware normalizing flow*. This equivariance introduces a physically motivated inductive bias, constraining the space of learnable coordinate transformations. The symmetry requirement does not imply that each individual input coordinate, or each individual normalizing-flow coordinate, must transform according to a single irreducible representation. Rather, each set of coordinates as a whole must transform as a reducible representation, i.e., as a direct sum of irreducible representations. For example, the two valence bond CH-stretches of H_2_CO do not individually transform according to any irreducible representation of the CNPI group *G*_4_ (isomorphic to *C*_2*v*_(*M*)). However, the symmetric and antisymmetric linear combination transform as *A*_1_ and *B*_1_, respectively, so the pair of CH-stretching coordinates collectively transform as *A*_1_ ⊕ *B*_1_.

### 2.3 Equivariant iResNet

To fulfill the aforementioned symmetry requirements, the normalizing-flow must be *G*-equivariant. This means that for all symmetry operations *g* ∈ *G*,

$$T_{g}\;f_{\theta }(r)=f_{\theta }(T_{g}r)$$

In other words, acting on the input coordinates with a symmetry operator is equivalent to acting on the output coordinates. Such an equivariant map can be constructed by applying a Reynolds average

$$q\to \mathrm{q̅}={\mathrm{f̅}}_{\theta }(r)=\frac{1}{\left|G\right|}\sum_{g\in G}T_{g}^{-1}f_{\theta }(T_{g}\cdot r)$$

The new change of coordinates **f̅**_θ_ can be verified to be *G*-equivariant, by applying a symmetry operator $T_{g^{\prime }}$ on both sides of the equation. By using group closure, *g*′ *g* = *g*″ with *g*″ ∈ *G*, and the fact that the inverse of the symmetry operators also belong to the group, one finds that

$${\mathrm{f̅}}_{\theta }(T_{g^{\prime }}\;r)=T_{g^{\prime }}\;{\mathrm{f̅}}_{\theta }(r)$$

This shows that the Reynolds average indeed produces a *G*-equivariant normalizing-flow map. However, this construction is not guaranteed to be invertible, and therefore it cannot be used to construct general coordinate transformations. A simple two-dimensional example illustrates the breakdown of the invertibility from the application of the Reynolds average. Consider the linear map

$$q=L(r)=\mathrm{Ar}=\left(\begin{array}{ll} 1 & 1\\ -1 & 0 \end{array}\right)\left(\begin{array}{l} r_{0}\\ r_{1} \end{array}\right)$$

This map is invertible since det­(**A**) = 1 ≠ 0. Let the symmetry group be *G* = {*E*, E*}, where *E* is the identity and E* is inversion acting as

$$\begin{array}{ll} T_{E}\cdot r & =\left(\begin{array}{ll} 1 & 0\\ 0 & 1 \end{array}\right)\left(\begin{array}{l} r_{0}\\ r_{1} \end{array}\right)\\ T_{E^{*}}\cdot r & =\left(\begin{array}{ll} 1 & 0\\ 0 & -1 \end{array}\right)\left(\begin{array}{l} r_{0}\\ r_{1} \end{array}\right) \end{array}$$

Each group element is an involution, i.e., *g* ^–1^ = *g*. The Reynolds-averaged linear map is

$$\begin{array}{lll} & \mathrm{L̅}(r)=\frac{1}{2}\left(\left(\begin{array}{ll} 1 & 1\\ -1 & 0 \end{array}\right)+\left(\begin{array}{ll} 1 & 0\\ 0 & -1 \end{array}\right)\left(\begin{array}{ll} 1 & 1\\ -1 & 0 \end{array}\right)\left(\begin{array}{ll} 1 & 0\\ 0 & -1 \end{array}\right)\right)\left(\begin{array}{l} r_{0}\\ r_{1} \end{array}\right)\\ & =\left(\begin{array}{ll} 1 & 0\\ 0 & 0 \end{array}\right)\left(\begin{array}{l} r_{0}\\ r_{1} \end{array}\right)=\mathrm{A̅}r & \end{array}$$

This map is no longer invertible since det­(**A̅**) = 0. Hence, the construction of symmetry-aware normalizing flows needs to be carefully adjusted, such that the Reynolds average cannot break the invertibility of the transformation.

We model the normalizing flow by an iResNet.^32^ An iResNet is constructed with a residual block structure, where the input of each block is the output of the previous one,

$$r_{k+1}=r_{k}+h_{k}(r_{k};\theta )=F_{k}(r_{k};\theta )$$

where *h_k_* (**r**_*k*_; θ) is a residual function modelled by a multilayer perceptron that depends on a subset of the parameters θ. The map *F_k_* (**r**; θ) is guaranteed to be invertible if *h_k_* has a Lipschitz constant smaller than 1. This condition is enforced by normalizing the spectral norm of the weight matrices and by using activation functions with Lipschitz constants ≤ 1. The complete iResNet transformation with *K* residual blocks can be written using composition as,

$$f_{\theta }(r)=\left(F_{K}◦L_{K}◦F_{K-1}◦L_{K-1}\;◦\;...\;◦\;F_{1}\right)\;(r)$$

where *L_k_* denotes an unrestricted linear transformation that does not couple different coordinates. The complete transformation, *f*_θ_, is constructed exclusively using composition. Function composition is an operation that produces equivariant functions out of equivariant functions. Therefore, to ensure equivariance of the iResNet, it suffices to ensure the equivariance of each residual function *h_k_* and each linear transformation *L_k_*.

To make the residual functions *h_k_* equivariant, we replace them with their Reynolds-averaged version,

5 $${\mathrm{h̅}}_{k}(r)=\frac{1}{\left|G\right|}\sum_{g\in G}T_{g}^{-1}h_{k}(T_{g}\cdot r)$$

The symmetry operators $T_{g}$ are orthogonal and their action preserves distances. Therefore, the Reynolds averaging over points on an orbit is non-expansive, ensuring that Lip­(*h̅*_*k*_) ≤ Lip­(*h_k_*). This proves that if the original iResNet map is invertible, the equivariant construction of the iResNet is also invertible.

For *L_k_* (**r**) = **α**_*k*_ **r** + **β**_*k*_, where **α**_*k*_ is a positive-definite diagonal matrix and **β**_*k*_ is a vector, we also use the Reynolds averages,

$$\begin{array}{ll} {\mathrm{L̅}}_{k}(r) & =\frac{1}{\left|G\right|}\sum_{g\in G}\left[T_{g}^{-1}\alpha_{k}T_{g}r+T_{g}^{-1}\beta_{k}\right]\\ & ={\mathrm{\alpha ̅}}_{k}r+{\mathrm{\beta ̅}}_{k} \end{array}$$

The transformation of **β̅**_*k*_ is an invariant Reynolds average that makes **β̅**_*k*_ invariant with respect to *g*. The positive-definite diagonal restriction of **α**_*k*_ guarantees that **α̅**_*k*_ is also a positive-definite diagonal matrix, which ensures its invertibility.

The output domain of the iResNet is unrestricted, and we use a wrapper such as, e.g., a hyperbolic tangent function, on the output of the inverse transformation (**q** → **r**) to squeeze the domain to [−1, 1] in each dimension. This domain is then mapped to the required coordinate domain by a linear transformation. The full normalizing-flow construction is a diffeomorphism, ensuring that the augmented basis is also a basis. Additional details on the iResNet architecture can be found in Saleh et al.^35^ and Vogt et al.^36^

Before proceeding, it is useful to place the present iResNet construction in the broader context of symmetry-aware invertible neural networks. In recent years, a number of normalizing-flow architectures have been proposed in which equivariance with respect to a prescribed symmetry group is enforced directly at the level of the invertible map, often by restricting the admissible network structure or by symmetrizing the transformation itself. Our approach may be viewed as a member of this general class of symmetrized iResNet constructions, specialized to discrete PI groups. As a representative example from this broader literature, Garcia Satorras et al.^48^ introduces equivariant normalizing flows for continuous Euclidean symmetry groups, providing a useful point of comparison.

### 2.4 Calculation Details

The underlying *a priori* basis was constructed as a direct-product of univariate Hermite basis functions. To control the size of the truncated direct-product basis, we used the polyad truncation 2­(*n*_1_ + *n*_2_ + *n*_3_) + *n*_4_ + *n*_5_ + *n*_6_ ≤ *P*_max_ for H_2_CO and 4­(*n*_1_ + *n*_2_ + *n*_3_) + 2­(*n*_4_ + *n*_5_) + *n*_6_ ≤ *P*_max_ for NH_3_, where *n_i_* denotes the Hermite basis function indices for coordinate *i*. The optimizations were performed for the first 100 lowest-energy states with *P*_max_ = 9 (1176 basis functions) for H_2_CO and *P*_max_ = 16 (1190 basis functions) for NH_3_. The calculations of H_2_CO and NH_3_ were performed with the PESs reported in Al-Refaie et al.^49^ and Polyansky et al.,^50^ respectively.

The Hamiltonian matrix elements were calculated using a Smolyak (sparse) quadrature constructed from Hermite quadratures. This structure creates a weighted sum of direct-product quadrature grids. The Smolyak grid was truncated using a total polynomial degree of 26/48 (H_2_CO/NH_3_) with the same polyad truncation used to control the size of the truncated direct-product basis. This creates a grid of 245,103/199,317 points. For H_2_CO, the CNPI group is abelian (commutative) and contains the symmetry operations *G*_4_ = {*E*, (12), E*, (12)*} and the irreducible representations *A*_1_, *A*_2_, *B*_1_, and *B*_2_. For NH_3_, the CNPI group is non-abelian and contains the symmetry operations *G*_12_ = {*E*, (23), (123), E*, (23)*, (123)*} and the irreducible representations $A_{1}^{\prime }$ , $A_{2}^{\prime }$ , *E*′, $A_{1}^{\prime \prime }$ , $A_{2}^{\prime \prime }$ and *E*″. The *G*_4_ PI group is isomorphic to *C*_2*v*_(*M*) and *G*_12_ is isomorphic to *D*_3*h*_(*M*). The effect of the *G*_12_ symmetry operations on the employed coordinates for NH_3_ can be found in Yurchenko et al.^51^

In Figure 1, the input internal angular coordinates for H_2_CO and NH_3_ are shown. The radial coordinates are the bond lengths. For NH_3_, the angles β_12_, β_13_ and β_23_ are defined by projecting the bond-angles α_12_, α_13_, and α_23_, respectively, onto the plane perpendicular to the trisector vector. These angles are then combined to the symmetry-adapted combinations $s_{4}=(2\beta_{23}-\beta_{13}-\beta_{12})/\sqrt{6}$ and $s_{5}=(\beta_{13}-\beta_{12})/\sqrt{2}$ . The last angular coordinate is ρ, which measures the angle between the trisector vector and the NH-bonds. The hyperbolic tangent wrapper used to map the output domain of the iResNet does not preserve the rotational (*C*_3_) symmetry of the angular coordinates (*s*_4_, *s*_5_). Therefore, we instead applied a radial hyperbolic tangent wrapper in the (*s*_4_, *s*_5_)-plane,

$$\left({\mathrm{s̃}}_{4},{\mathrm{s̃}}_{5})=\frac{\tanh\;\left(r_{s}\right)}{r_{s}}(s_{4},s_{5}\right)$$

where $r_{s}=\sqrt{s_{4}^{2}+s_{5}^{2}}$ . This mapping squeezes the domain to a unit disk while preserving the rotational symmetry in the (*s*_4_, *s*_5_)-plane. The subsequent linear transformation, also applied to the other coordinates, maps this unit disk to a disk of radius $\sqrt{6}\pi /3$ . The original transformation from β_12_, β_13_, β_23_ to *s*_4_, *s*_5_ produces a hexagonal domain with a circumradius of $\sqrt{6}\pi /3$ . Thus, the transformed disk contains the hexagonal domain, but also extends slightly beyond it. However, as the potential rises steeply near the extreme angular configurations, the amplitude of the wavefunctions is strongly suppressed there, and we did not experience numerical issues originating from the mapping itself.

**Figure 1:**
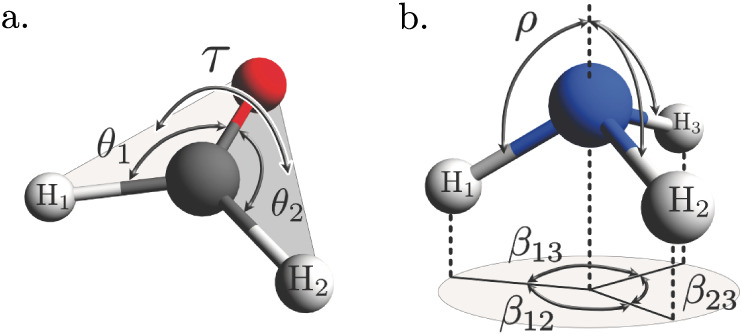
Angular coordinates for H_2_CO (a) and NH_3_ (b). Reproduced from ref 52. Available under the CC BY 4.0 license. Copyright 2025 Yachmenev et al.

To construct symmetry-adapted basis functions, the action of the symmetry operators on the direct-product basis were formulated. Here, we briefly outline the procedure used for NH_3_. For the stretching coordinates, the symmetry operators act as permutation matrices on the basis indices, (*n*_1_, *n*_2_, *n*_3_). The bending coordinates (*s*_4_, *s*_5_) span a two-dimensional space, in which the symmetry operators act as planar rotations or reflections. The permutation operators mix states with the same value of *n*_4_ + *n*_5_, and their action was represented by Wigner small-*d* matrices of rank (*n*_4_ + *n*_5_)/2 with rotation angles of 0 or ±2π/3. Inversion affects neither the stretching nor the bending coordinates. The dihedral (umbrella) coordinate was defined on the domain [−π, π], and it changes sign under odd permutations and under inversion. The corresponding basis functions are symmetry-adapted by construction: they are even under sign change for even values of *n*_6_, and odd under sign change for odd values of *n*_6_.

With the actions of the symmetry operators on the direct-product basis indices (*n*_1_, ..., *n*_6_) defined, we first partitioned the full set of basis indices (*N* × 6) into orbits closed under the *G*_12_ PI group. For each orbit, reducible representation matrices, *D*(*g*), were constructed from direct products of permutation matrices for (*n*_1_, *n*_2_, *n*_3_), Wigner small-*d* matrices for (*n*_4_, *n*_5_), and parity factors for *n*_6_. Symmetry-adapted linear combinations were obtained by applying the projection operator in eq 4 for each irreducible representation. The rows of each projector were orthonormalized using singular value decomposition to produce a set of symmetry-adapted linear combinations transforming as that irreducible representation. The final orthogonal transformation matrix **U**, used to symmetrize the Hamiltonian **H** → **UHU** ^*T*^, was assembled by concatenating symmetry-adapted linear combinations from all orbits and all irreducible representations.

The employed iResNet architecture consisted of five residual blocks. Each block contained two hidden layers consisting of 8 units and an output layer with the number of units matching the number of coordinates. The spectral norm of the weight matrices was normalized by capping each singular value to 0.9. The loss function (eq 3) was optimized with the Optax Adam optimizer with a learning rate of 0.001, β_1_ = 0.9, β_2_ = 0.999, ϵ = 10^–8^, and ϵ̅ = 0.0.^53^

### 2.5 Coordinate Variability

We analyzed the optimized normalizing-flow coordinates to quantify how each output coordinate depends on the underlying input coordinates. For a given configuration **r**_*g*_, the local sensitivity of the transformed coordinates **q** = *f*_θ_(**r**) to variations in **r** is described by the Jacobian matrix **J**,

$$J_{ij}=\frac{\partial f_{\theta ,i}}{\partial r_{j}}{|}_{r_{g}}$$

The Jacobian can be expressed either as a function of **r** or, equivalently, as a function of **q**. If a Jacobian matrix element *J*_*ij*_ is zero at all points, then the output coordinate *q_i_* and the input coordinate *r_j_* are completely independent of one another.

To obtain a global and coordinate-wise measure of these local sensitivities, we employ the derivative-based global sensitivity measure (DBGSM). The DBGSM aggregates the magnitude of the Jacobian elements over configuration space, yielding a scalar measure of how strongly each input coordinate influences each output coordinate. Like the Jacobian, the DBGSM is naturally organized as a matrix with output coordinates as rows and input coordinates as columns. It is defined as the dimensionless quantity

$$D_{ij}=\frac{\Delta r_{j}}{\Delta q_{i}^{2}}\int |J_{ij}(q)|\;dq$$

where Δ*r*_*j*_ and Δ*q*_*i*_ are the respective grid ranges, introduced to treat coordinates with different units on equal footing. To make input coordinate contributions comparable across different output coordinates, we normalized the rows of **D** to one.

### 2.6 Decay Behavior

The numerical convergence of augmented bases of the form eq 1, with the *a priori* basis chosen as Hermite functions, was recently studied in Saleh.^45^ It was shown that, when the normalizing flow is trained to minimize the approximation error, it learns a coordinate transformation that causes the target states, when expressed in the transformed coordinates, to exhibit asymptotic behavior similar to that of Hermite functions.

To analyze the decay behavior of the computed eigenstates, we calculated the integrals

$$L_{m}(s)=\int {\mathrm{\Psi ̃}}_{m}^{*}(q)\left(\prod_{i=1}^{6}|q_{i}{|}^{s_{i}}\right){\mathrm{\Psi ̃}}_{m}(q)\;dq$$

where **s** = (*s*_1_, ..., *s*_6_) is a multi-index and six is the number of vibrational coordinates for both H_2_CO and NH_3_. The indices *s_i_* were restricted to positive integers.

Rather than considering individual multi-indices, we group them according to their total order $S=\overset{6}{\underset{i=1}{\sum }}s_{i}$ . For a fixed value of *S*, we define the set

$$S_{S}=\left\{s=(s_{1},\;...,\;s_{6})\;|\;\overset{6}{\underset{i=1}{\sum }}s_{i}=S\right\}$$

which contains all multi-indices with the same total order. We then define the averaged quantity

6 $$I_{m}(S)=\frac{1}{\left|S_{S}\right|}\sum_{s\in S_{S}}L_{m}(s)$$

The rate at which *I_m_* (*S*) changes with *S* provides a measure of the decay of the *m*th eigenstate. If Ψ̃_*m*_ is localized, *I_m_* (*S*) will be small for larger values of *S*, compared to that of delocalized states.

To characterize the average decay behavior of the subset of the lowest 100 energy eigenstates transforming according to a given irreducible representation Γ, we further averaged *I_m_* (*S*) over all states belonging to that representation. Specifically, we define

7 $$I^{\Gamma }(S)=\frac{1}{\left|M_{\Gamma }\right|}\sum_{m\in M_{\Gamma }}I_{m}(S)$$

where $M_{\Gamma }=\left\{m|P_{g}{\mathrm{\Psi ̃}}_{m}=\Gamma (g){\mathrm{\Psi ̃}}_{m}\;\forall g\in G\right\}$ denotes the set of indices corresponding to eigenstates that transform according to Γ.

## 3 Results and Discussion

### 3.1 H_2_CO

In Figure 2, we present the convergence of the lowest 100 approximate vibrational energies for H_2_CO as a function of the number of epochs. The convergence of a single energy level *m* is defined as the absolute difference between the approximate energy *Ẽ*_*m*_ and its reference value $E_{m}^{\mathrm{ref}}$ :

$$\Delta E_{m}={Ẽ}_{m}-E_{m}^{\mathrm{ref}}$$

The reference energies were obtained from a converged calculation using the symmetry-aware iResNet architecture with 18876 basis functions (*P*_max_ = 17).

**Figure 2:**
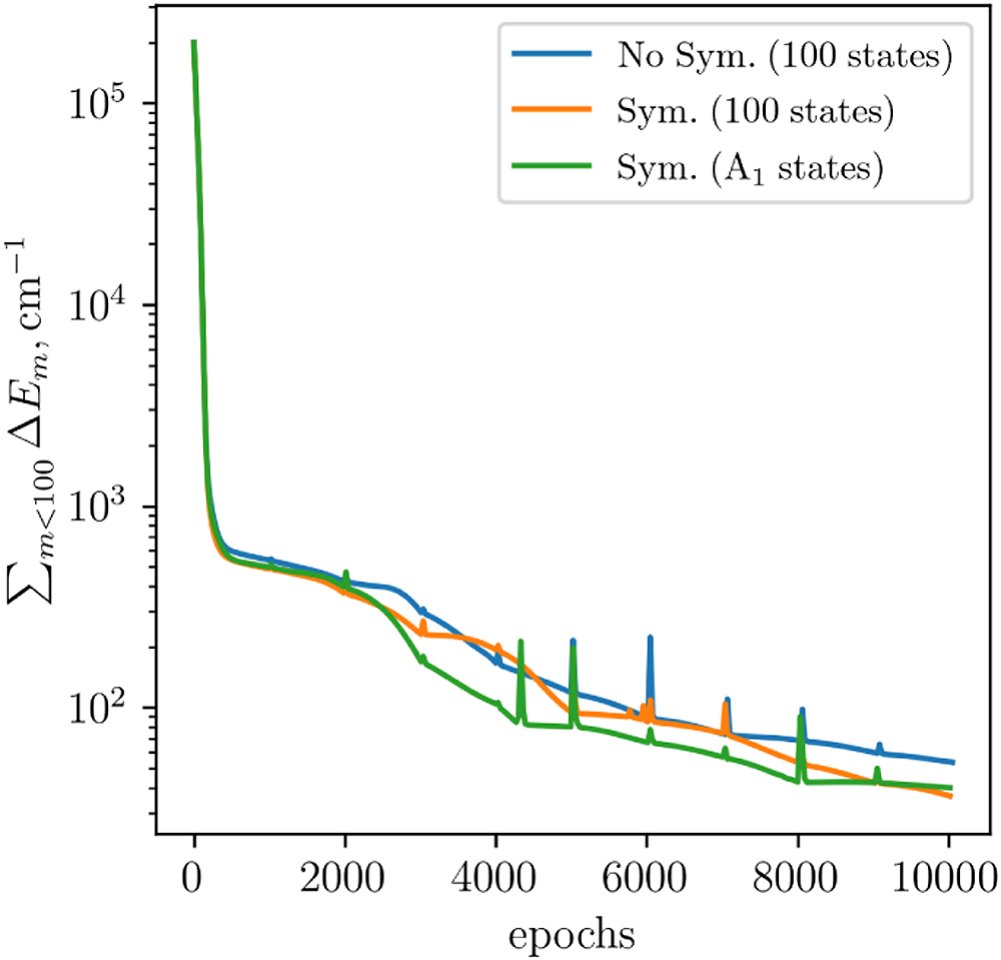
Total convergence of the lowest 100 approximate vibrational energies of H_2_CO as a function of number of epochs. Reference results were obtained from a converged calculation using symmetry-adapted normalizing-flow coordinates with 18876 basis functions (*P*_max_ = 17). In all three cases, the figure shows the same quantity: total convergence of the lowest 100 energies. The blue and orange curves correspond to optimizations performed without and with explicit symmetry enforcement, respectively. For both curves, the loss function was defined as the sum of the lowest 100 approximate energies. The green curve shows the total convergence of the same sum of the lowest 100 energies, but for an optimization in which the loss function was instead defined as the sum of the 38 lowest-energy *A*_1_ states.

To quantify the overall agreement across all 100 states, we define the total convergence as the sum of the convergences of the individual energy levels (∑_*m*<100_ Δ*E_m_*). Results are shown both with (orange curve) and without (blue curve) imposing symmetry on the normalizing-flow coordinates. The curves are generated using linear interpolation between points spaced at 30 epoch intervals. The sharp spikes in the curves every 1000th epoch are due to reinitialization of the Adam optimizer. For both optimizations, the loss function was defined as the sum of the lowest 100 approximate energies.

As shown in Figure 2, enforcing symmetry in the normalizing-flow coordinates has a positive impact on the overall convergence behavior. This indicates that the constraints imposed by the symmetry-restricted architecture do not compromise the quality of the optimized coordinates. The green curve presents the results obtained by optimizing the normalizing-flow coordinates using only the 38 lowest-energy *A*_1_ states (38 of the 100 lowest-energy states belong to the *A*_1_ representation). Notably, optimizing the coordinates for this *A*_1_ subset yields faster convergence of the first 100 approximate energies compared to using the sum over all 100 energies as the loss function.

The training time was evaluated for H_2_CO with *P*_max_ = 9 (1176 basis functions) across four distinct cases: (A) without symmetry on the full (non-symmetrized) grid, (B) with symmetry enforced on the same grid, (C) with symmetry on a symmetry-reduced integration grid, and (D) with symmetry on the reduced grid restricted to the A1 states. All calculations were performed on a node equipped with two NVIDIA Tesla V100-SXM2 GPUs. Enforcing symmetry introduces an additional computational overhead due to Reynolds averaging of the residual blocks and linear transformations. This increases the time per epoch from approximately ∼4.1 s in case (A) to ∼8.2 s in case (B). However, the computational overhead is offset by the symmetry reduction of the integration grid, leading to ∼2.4 s per epoch in case (C). A further reduction is achieved when restricting the optimization to *A*_1_ states, due to the smaller Hamiltonian matrix to construct and diagonalize. This leads to ∼2.1 s per epoch for case (D).

In Vogt et al.^36^ we demonstrated that normalizing-flow coordinates optimized to minimize the approximate energies effectively shift the average density of the eigenfunctions to better match that of the basis functions, here Hermite functions. Although the average density distribution is expected to be largely similar across irreducible representations, this effect alone cannot explain the improved convergence behavior observed when optimizing for the *A*_1_ subset. Instead, we argue that it can be understood from the asymptotic behavior of the eigenstates expressed in **q**.

Classical approximation theory of Hermite functions states that the optimal convergence rate is achieved when the target functions—here, the eigenstates—exhibit asymptotic behavior similar to the exponential factor $e^{-q^{2}/2}$ of the Hermite functions.^54^ Similarly, it was demonstrated that normalizing-flow coordinates optimized to minimize the approximate energies effectively adjust the asymptotic behavior of the eigenstates to match that of the Hermite functions.^45^ If the asymptotic behavior of eigenstates is similar across irreducible representations, optimizing the coordinates using only the *A*_1_ subset would preserve the key features required to align the asymptotic behavior of the full set of 100 wavefunctions with that of the Hermite functions.

To test this hypothesis, we evaluated the integrals defined in eq 7 using the optimized coordinates computed *via* the symmetry-aware normalizing-flow, for *S* = 1, 2, ..., 8. For each irreducible representation, we fitted a straight line to log_10_(*I* ^Γ^(*S*)) as a function of *S*. The resulting slopes and intercepts are summarized in Table 1.

**Table 1:** Slopes and Intercepts Obtained from Linear Fits to log_10_(*I* ^Γ^(*S*)) as a Function of *S* for Different Irreducible Representations

Γ	Slope	Intercept
*A*_1_	0.175 ± 0.005	–0.093 ± 0.02
*A*_2_	0.176 ± 0.004	–0.083 ± 0.02
*B*_1_	0.175 ± 0.005	–0.093 ± 0.02
*B*_2_	0.176 ± 0.004	–0.084 ± 0.02

The extracted slopes are nearly identical across all irreducible representations, with values clustered around 0.175, and similarly consistent intercepts of approximately −0.085. This near equality of the averaged decay rates supports our interpretation of why optimizing to the *A*_1_ subset yields nearly identical optimal coordinates to those obtained for the full set of 100 lowest energy states. We did not observe a strong correlation between the decay behavior of individual states and their energies, indicating that the conclusions drawn here apply only for a large enough subset of states.

For H_2_CO, the assigned irreducible representations of the 100 lowest-energy states are identical to those computed with TROVE using the same PES.^49^ The TROVE energies were computed with a contracted basis consisting of 7641 basis functions. The mean deviation from our converged energies are −0.5 cm^–1^, with all energies computed in this work being slightly lower. The discrepancies are due to both basis convergence and the use of Taylor expansions of the kinetic and potential energy operators in the TROVE calculations.

In Figure 3, we present the convergence of the H_2_CO vibrational energies with respect to the number of basis functions. The energies are computed using both symmetry-aware iResNet and a symmetrized linear parametrization of the input valence-bond coordinates as coordinate transformations. The resulting energies are grouped according to their associated irreducible representations. Results are shown for calculation using 1176 and 5544 basis functions, corresponding to *P*_max_ = 9 and *P*_max_ = 13, respectively. The normalizing-flow parameters were optimized using 1176 basis functions and subsequently transferred without any adjustments to the calculations employing 5544 basis functions. Basis-set convergence curves for additional values of *P*_max_ are provided in the supplementary repository. The reference energies are converged to an accuracy exceeding that of the presented results by more than an order of magnitude.

**Figure 3:**
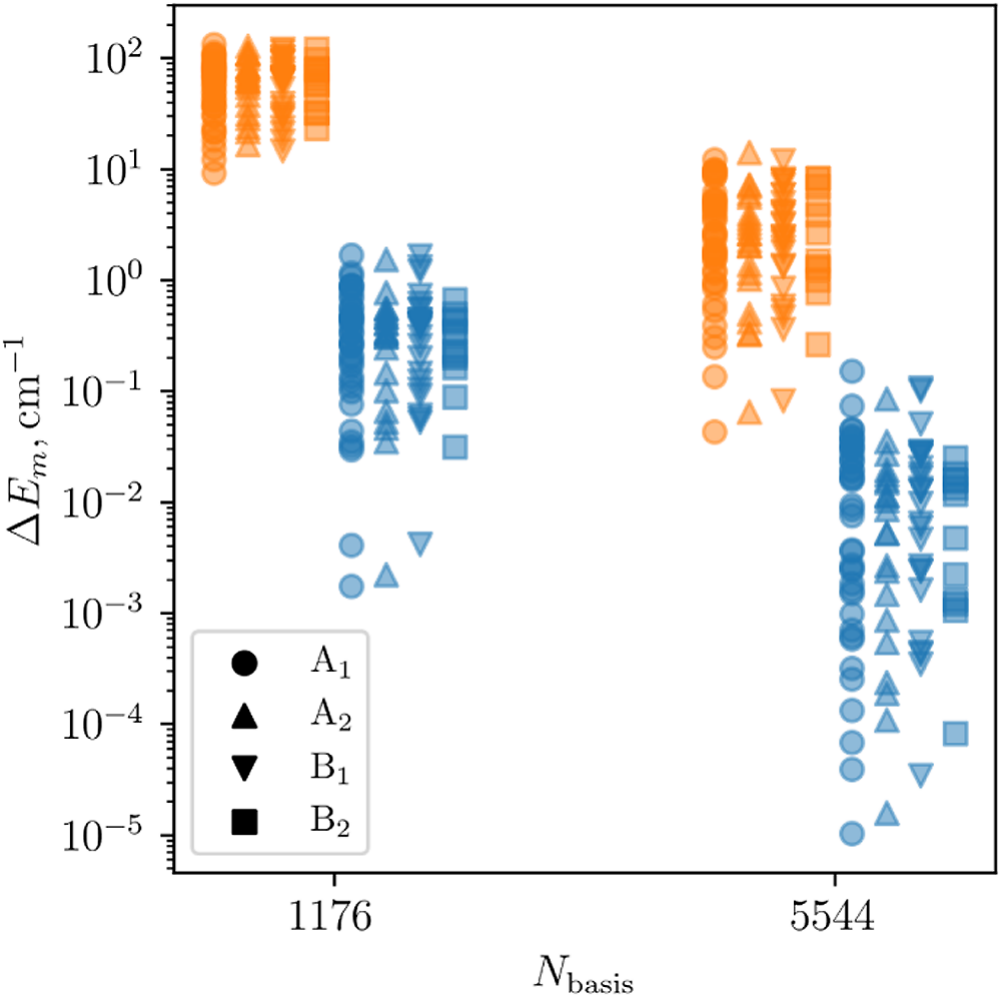
Convergence of H_2_CO vibrational energies with respect to the number of Hermite basis functions, *N*_basis_. Orange: results obtained using a symmetrized linear parametrization of the input valence-bond coordinates. Blue: results obtained using symmetry-aware iResNet. The parameters were optimized using 1176 basis functions and subsequently transferred without any adjustments to the calculations using 5544 basis functions. Reference energies were computed with the same normalizing-flow coordinates, with 18876 basis functions (*P*_max_ = 17). The data points are offset along the horizontal axis for visual clarity.

As seen in Figure 3, the energies computed for different irreducible representations exhibit very similar convergence behavior for both coordinate choices, indicating that the underlying optimal (linear or non-linear) coordinate transformation is largely shared across irreducible representations. The energies computed with normalizing-flow coordinates converge significantly faster than those computed with the linearly optimized valence-bond coordinates. In fact, the normalizing-flow calculations with only 1176 basis functions achieve better convergence than that of the valence-bond coordinates using 5544 basis functions. This demonstrates that employing optimized coordinates can significantly reduce the computational cost required to reach a given accuracy.

In Figure 4, we show single-coordinate displacements from the equilibrium geometry for the normalizing-flow coordinates of H_2_CO optimized using a symmetry-aware iResNet. For visual purposes, an embedding that fixes the carbon atom and the CO-direction was employed. Although these coordinates are more abstract than conventional choices of curvilinear coordinates, in part due to their non-geometrical formulation, the resulting displacements nevertheless map cleanly onto common notions of molecular motion. Near the equilibrium geometry, the normalizing-flow coordinates can be interpreted as linear combinations of the underlying valence-bond coordinates.

**Figure 4:**
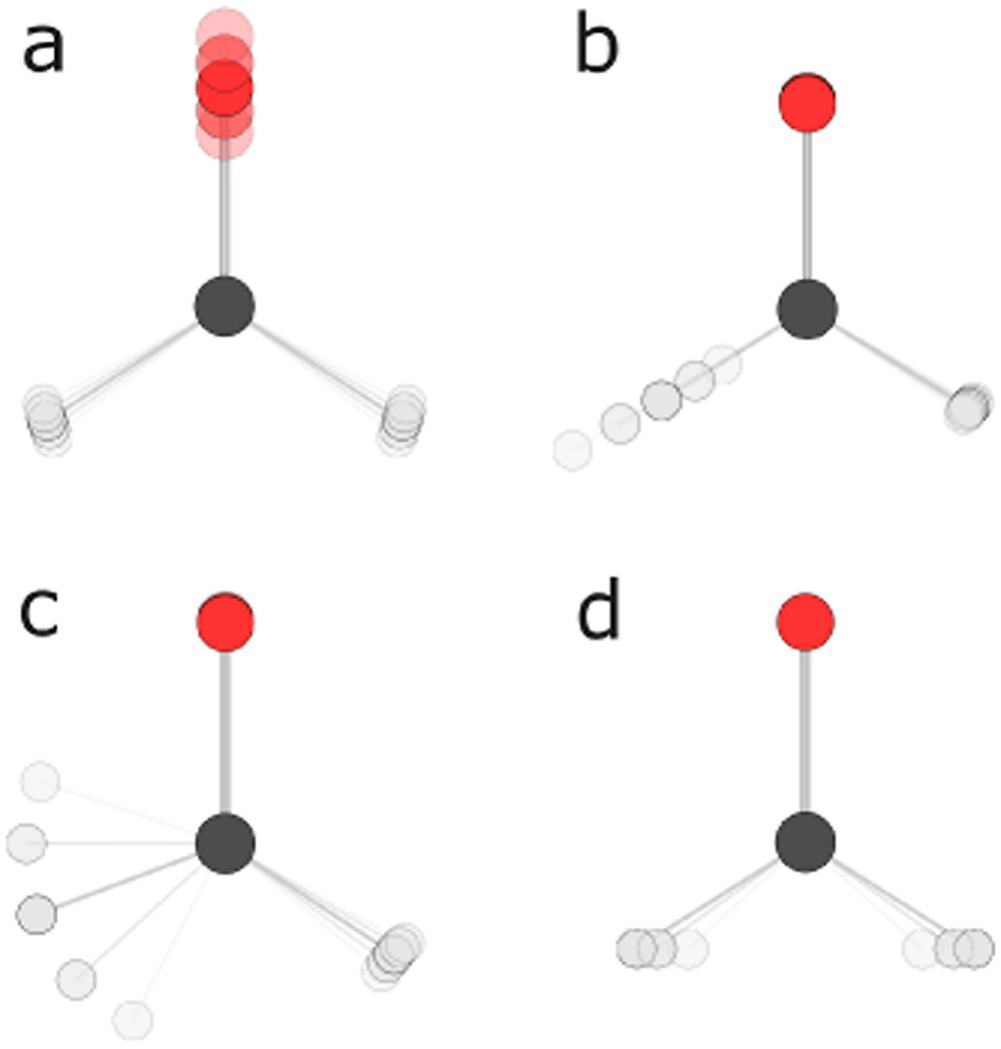
Optimized normalizing-flow coordinates for H_2_CO, obtained by training a symmetry-aware iResNet and visualized by displacing individual coordinates from the equilibrium configuration. (a) *q*_1_ resembles a CO-stretch; (b) *q*_2_ resembles a CH-stretch (*q*_3_ is the mirror image of *q*_2_ due to molecular symmetry); (c) *q*_4_ resembles a COH-bend (*q*_5_ is the mirror image of *q*_4_ due to molecular symmetry); (d) *q*_6_ resembles the dihedral angle.

In Saleh et al.^35^ we demonstrated that, when using a direct-product basis, the normalizing-flow coordinates enhance mode separability to improve basis convergence. Improved mode separability also facilitates the assignment of approximate quantum numbers from projections onto single-mode (one-dimensional) eigenfunctions. A natural objection to employing normalizing-flow coordinates is that their more complex and non-geometrical formulation may obscure the physical meaning of the vibrations they represent. However, an analogous situation arises with normal coordinates, which are also mathematical abstractions relative to internal valence coordinates, yet are commonly assigned physical labels based on visual inspections of their displacements around a reference geometry. The normalizing-flow displacements shown in Figure 4 likewise yield physically interpretable vibrational motions. In fact, since these coordinates yield a more separable Hamiltonian, in some sense, they provide a learned representation of the intrinsic vibrational motions.

In Figure 5, we present the normalized DBGSM matrix comparing the optimized normalizing-flow coordinates for H_2_CO—obtained by training a symmetry-aware iResNet—with the input valence-bond coordinates. The matrix has relatively small off-diagonal values (note the logarithmic scale), showing that the main contribution to each normalizing-flow coordinate is the associated input coordinate. This is expected based on the block structure of the normalizing flow *F̅*_*k*_(**r**_*k*_; θ) = **r**_*k*_ + *h̅*_*k*_(**r**_*k*_; θ). The structure of the matrix reflects the imposed equivariance *via* the symmetry-aware iResNet. For example, the values associated with *q*_1_ and the two CH-bond stretches (*r*_2_ and *r*_3_) are identical, and the coupling of *q*_2_ with *r*_3_ mirrors that of *q*_3_ with *r*_2_, as required by the permutation symmetry of the two hydrogen atoms. The dihedral angle *τ* transforms as the *A*_2_ irreducible representation, which is not contained in the symmetry species of the other coordinates. Therefore, no symmetry-adapted linear combination can couple *τ* to the other valence-bond coordinates. Because the symmetry-aware normalizing-flows transformation is non-linear, variations of the coordinates *q*_1_ to *q*_5_ can be seen due to variations of τ. Identically, the non-linear mapping enables the variation of *q*_6_ as a function of the rest of the valence-bond coordinates.

**Figure 5:**
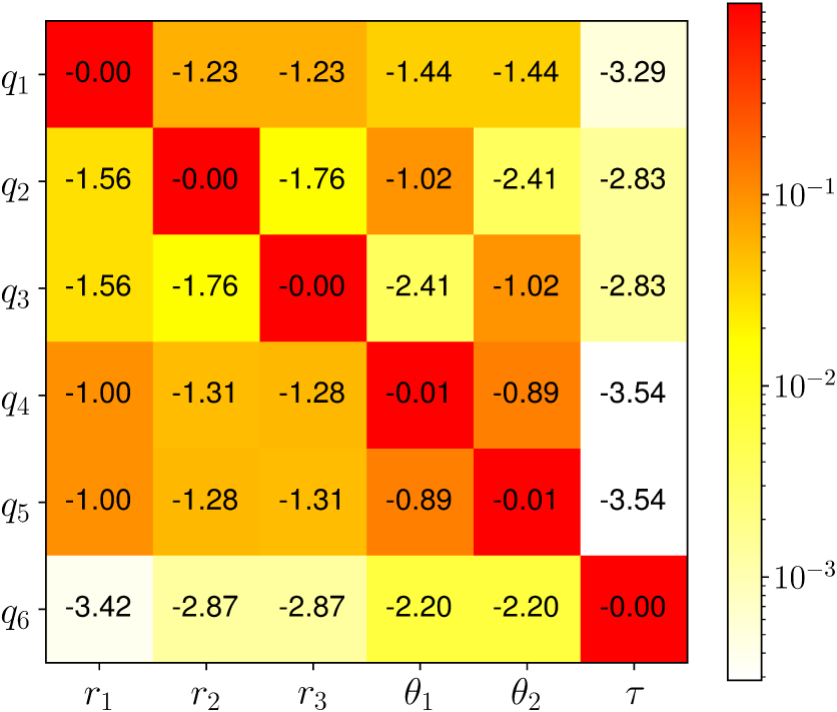
Normalized derivative-based global sensitivity measure matrix for the normalizing-flows coordinates for H_2_CO obtained *via* symmetry-aware iResNet. The matrix shows the variance of each input coordinate with respect to variations in each normalizing-flow coordinate. The log_10_ value of the computed matrix elements is displayed on each entry. The white-to-red colormap indicates weak to strong coupling. The exact equivariance of the normalizing-flow coordinates is apparent from the block structure.

### 3.2 NH_3_

Calculations of accurate energy levels of NH_3_ are more challenging than for the semi-rigid H_2_CO molecule. The increased complexity arises due to its large-amplitude umbrella motion that connects the two energetically equivalent configurations, and from the non-commutitative character of the CNPI group *G*_12_. Given the apparent differences between H_2_CO and NH_3_, one might anticipate clear differences in the corresponding results.

In Figure 6, we present the total convergence of the lowest 100 approximate energies for NH_3_ as a function of the number of epochs, i.e., nonlinear optimization steps. Results are shown both with (orange curve) and without (blue curve) enforcing symmetry on the normalizing-flow coordinates. For both optimizations, the loss function was defined as the sum of the lowest 100 approximate energies. Reference energies were computed using the symmetry-aware iResNet as a coordinate transformation and 13266 basis functions (*P*_max_ = 28).

**Figure 6:**
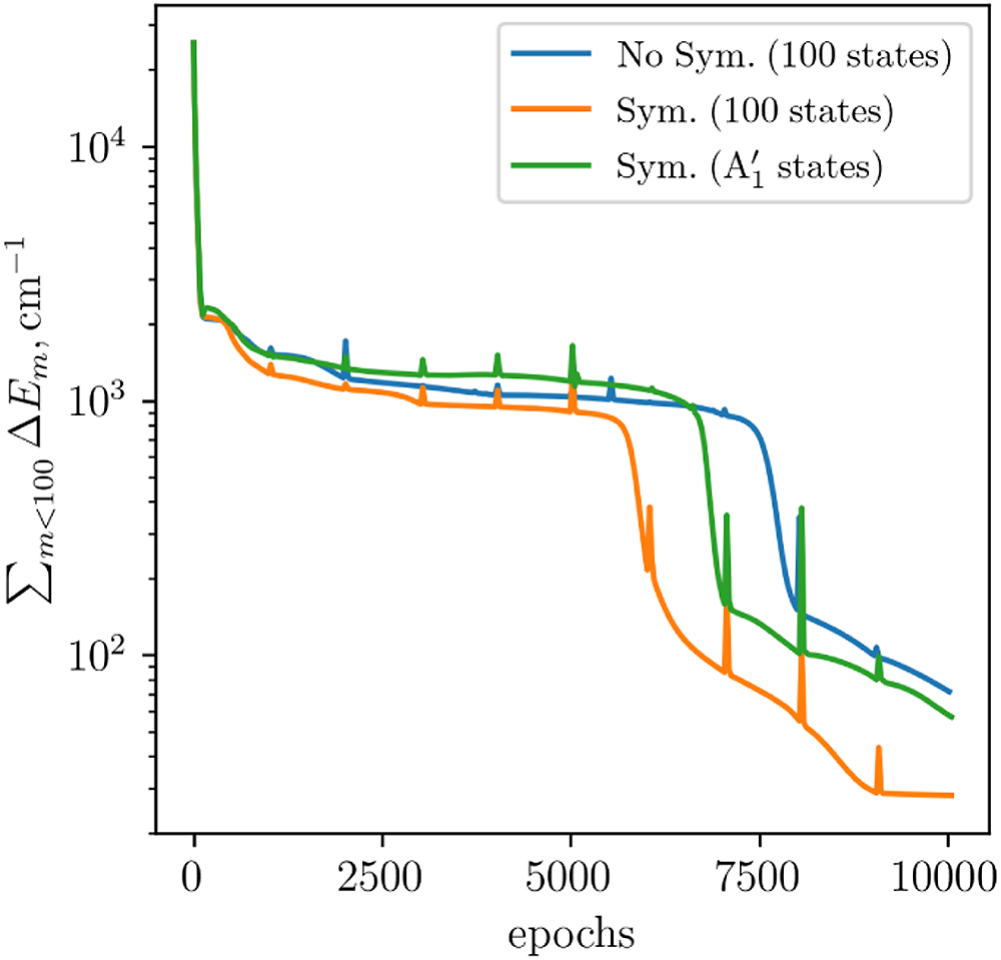
Total convergence of the lowest 100 approximate energies for NH_3_ as function of number of epochs. Reference energies were computed with the converged symmetry-aware iResNet and 13266 basis functions (*P*_max_ = 28). In all three cases, the figure shows the same quantity: total convergence of the lowest 100 energies. The blue and orange curves correspond to optimizations performed without and with explicit symmetry enforcement, respectively. For both curves, the loss function was defined as the sum of the lowest 100 approximate energies. The green curve shows the total convergence of the same sum of the lowest 100 energies, but for an optimization in which the loss function was instead defined as the sum of the lowest 17 $A_{1}^{\prime }$ states.

The green curve shows the results obtained when optimizing the symmetry-aware normalizing-flow using only the 17 lowest-energy $A_{1}^{\prime }$ states (17 out of the 100 lowest-energy states transform as $A_{1}^{\prime }$ ).

As shown in Figure 6, enforcing symmetry on the normalizing-flow coordinates has little influence on the convergence behavior during the first approximately 5700 epochs. Beyond this point, normalizing-flow coordinates obtained using the symmetry-aware iResNet exhibit significantly faster convergence, in contrast to the behavior observed for H_2_CO in Figure 2. The convergence obtained by optimizing to the symmetry-specific subset of states (green curve) is slower than that achieved with the symmetry-aware optimization for the full set of the 100 lowest-energy states, yet still faster than the corresponding optimization performed without imposing symmetry on the normalizing-flow coordinates. The results again demonstrate that the symmetry-restricted normalizing-flow architecture does not limit the quality of the optimized coordinates. The comparable $L_{\theta }^{100}$ values obtained for the optimization to the $A_{1}^{\prime }$ subset of states, after 10000 epochs, further supports the hypothesis that optimal coordinates are shared across irreducible representations.

In Figure 7, we present the convergence of the NH_3_ vibrational energies with respect to the number of basis functions. The energies are computed using optimized normalizing-flow coordinates obtained from a symmetry-aware iResNet, and with a symmetrized linear parametrization of the input NH_3_ coordinates. Results are shown for calculations with 1190 and 2982 basis functions, corresponding to *P*_max_ = 16 and *P*_max_ = 20, respectively. The coordinate parameters were optimized in the calculation with 1190 basis functions and subsequently transferred without any adjustments to the calculations employing 2982 basis functions.

**Figure 7:**
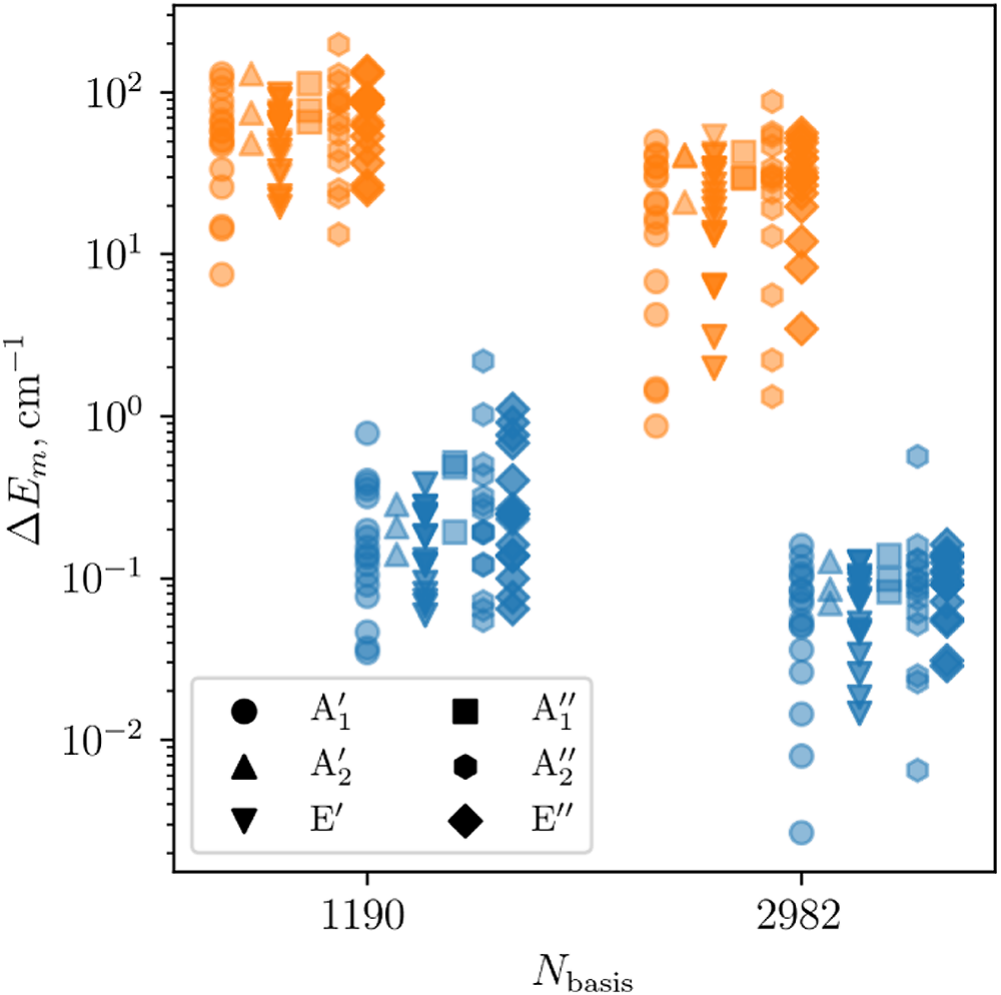
Convergence of NH_3_ vibrational energies with respect to the number of Hermite basis functions, *N*_basis_. Orange: results obtained using a symmetrized linear parametrization of the input coordinates. Blue: results obtained using normalizing-flow coordinates obtained *via* a symmetry-aware iResNet. The parameters were optimized using 1190 (Hermite) basis functions and subsequently transferred without any adjustments to the calculations using 2982 basis functions. Reference energies were computed with the same normalizing-flow coordinates, with 13266 basis functions (*P*_max_ = 28). The data points are offset along the horizontal axis for visual clarity.

As seen in Figure 7, the vibrational energies of NH_3_ show similar convergence for the different irreducible representations, as seen for H_2_CO in Figure 3. Again, the computed energies are significantly improved by using normalizing-flow coordinates obtained *via* a symmetry-aware iResNet, compared with the chosen input coordinates.

In Figure 8, we show single-coordinate displacements from the equilibrium geometry of the optimized normalizing-flow coordinates for NH_3_, obtained by training a symmetry-aware iResNet. For visual purposes, an Eckart embedding was employed with one of the minimum-energy configurations as the reference structure, followed by fixing the position of the nitrogen atom. As for H_2_CO (Figure 4), the normalizing-flow coordinates near one of the minimum-energy configurations map cleanly onto common notions of molecular motions. In this region near the reference configuration, the normalizing-flow coordinates again resemble the input coordinates.

**Figure 8:**
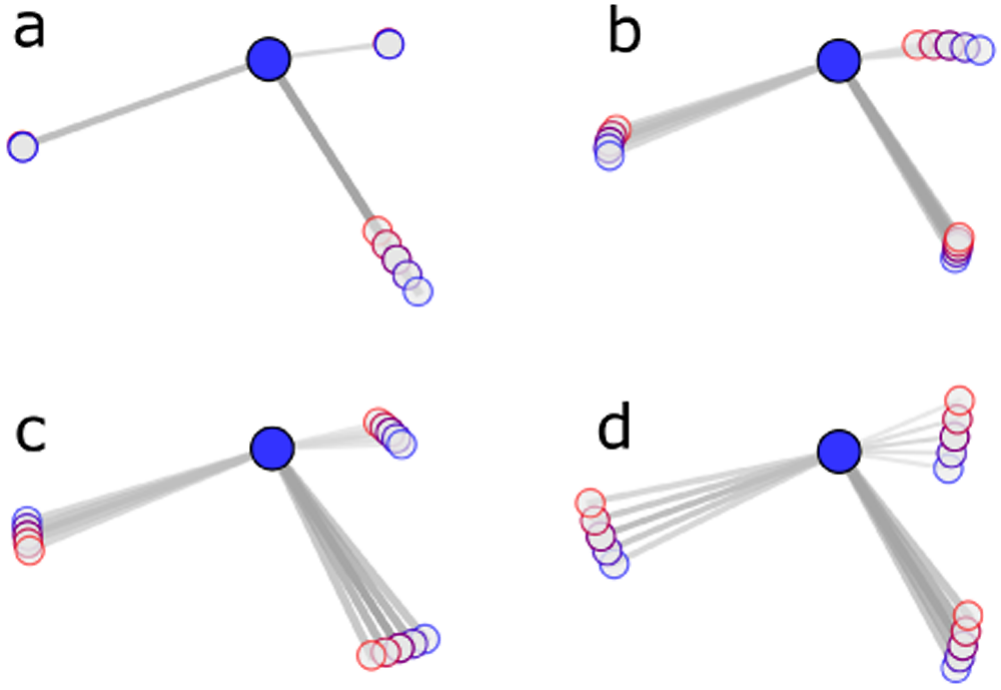
Optimized normalizing-flow coordinates for NH_3_, obtained by training a symmetry-aware iResNet and visualized by displacing individual coordinates from the equilibrium configuration. A blue-to-orange edge color on the hydrogen atoms indicates the displacement direction. (a) *q*_1_ resembles an NH-stretch (*q*_2_ and *q*_3_ are similar due to molecular symmetry); (b) *q*_4_ resembles the HNH-bending coordinate *s*_4_; (c) *q*_5_ resembles the HNH-bending coordinate *s*_5_; (d) *q*_6_ resembles the umbrella (dihedral) coordinate.

In Figure 9, we present the DBGSM matrix for the optimized normalizing-flow coordinates for NH_3_, obtained by training a symmetry-aware iResNet, in relation to the input coordinates. The dominant contribution to each normalizing-flow coordinate comes from its associated input coordinate, as reflected by the large diagonal elements and comparatively small off-diagonal elements. The stronger symmetry constraints imposed by the larger CNPI group of NH_3_, relative to that of H_2_CO, are evident from the repeated entries. As for H_2_CO, the non-linear nature of the transformation allows the normalizing-flow coordinate associated with the dihedral angle to vary as a function of the bond lengths. In Saleh et al.^35^ we showed that the normalizing-flow coordinate optimized for the angular Jacobi coordinate in HCN closely followed the minimum-energy path between the HCN and HNC isomers, naturally leading to a decoupling of the vibrational modes. If *q*_6_ for NH_3_ similarly follows the minimum-energy inversion path between the two equivalent configurations, a variation of the NH-bond lengths is expected along the path, since the optimized bond lengths at the inversion transition state are shorter by ∼0.015 Å.^55^

**Figure 9:**
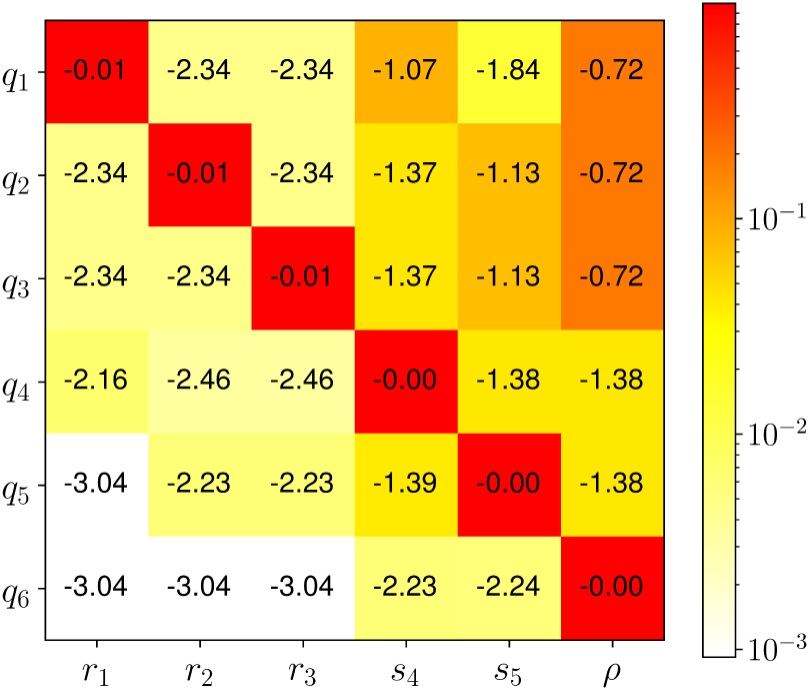
Normalized derivative-based global sensitivity measure matrix for the normalizing-flows coordinates for NH_3_ obtained *via* symmetry-aware iResNet. The matrix shows the variation of each normalizing-flows coordinate with respect to variations in each input coordinate. The log_10_ value of the computed matrix elements is displayed on each entry. The white-to-red colormap indicates weak to strong correlation. The exact equivariance of the normalizing-flow coordinates is apparent from the block structure of the matrix.

## 4 Conclusions

Normalizing flows offer a promising new route for advancing spectral methods through generalized coordinate optimization. In this and earlier work,^35,36^ we have demonstrated that normalizing-flow coordinates can substantially improve the accuracy and efficiency of variational vibrational calculations.

Unlike conventional geometrically defined coordinates, normalizing-flow coordinates do not rely on a fixed arbitrary construction. Instead, they can be tailored to the problem at hand through optimization and, with appropriate model and parameter choices, can reproduce traditional coordinates. Despite their promising potential, these new type of coordinates also introduce new challenges. The computational overhead of the optimization process can be significant, and in their initial formulation, the normalizing-flow transformations were not symmetry-aware—an arguably essential feature for optimal coordinate transformations.

In this work, we developed a symmetry-aware formulation of normalizing-flow coordinates. Symmetry was enforced by requiring the coordinate transformation to be equivariant with respect to discrete symmetries of the molecular symmetry group acting on the input coordinates. In this way, the normalizing-flow coordinates inherit the symmetry properties of the input coordinates. Equivariance of the normalizing-flow coordinates was accomplished through Reynolds averaging of the residual functions and of the linear transformations within the iResNet structure. This ensures exact equivariance while retaining expressivity of the normalizing-flow. We demonstrated exact equivariance and retention of expressivity in computations of vibrational energies of H_2_CO and NH_3_. This observation is consistent with previously reported results, which show that constraining the flexibility of neural networks to enforce expected system behavior can be beneficial.^56,57^

The similar convergence behavior of energy levels observed across irreducible representations suggests that the optimal coordinate mapping is shared across irreducible representations. Additional evidence comes from the comparable optimization behavior obtained when employing an alternative loss function based on energies associated with a single irreducible representation. This observation indicates the potential for computationally more efficient optimization procedures, which may extend the applicability of normalizing-flow coordinates to high-dimensional molecular systems.

The more abstract non-geometrical formulation of the normalizing-flow coordinates may seem at odds with physically intuitive descriptions of vibrational motion. However, as demonstrated for both H_2_CO and NH_3_, optimized normalizing-flow coordinates map cleanly onto common notions of molecular vibrations. At the same time, the enhanced separability of the Hamiltonian expressed in normalizing-flow coordinates indicates that the learned coordinates offer a more intrinsic representation of vibrational motions, compared with conventional coordinate choices.

## Data Availability

The data supporting the findings of this study are available within the article and through the following repository: https://github.com/robochimps/symm-flows. The code developed in this work is available at: https://github.com/robochimps/symm-flows.
